# Niemann-Pick type A disease with new mutation: a case report

**DOI:** 10.1186/s13256-022-03486-5

**Published:** 2022-07-27

**Authors:** Fatemeh Aghamahdi, Matineh Nirouei, Shahram Savad

**Affiliations:** 1 Pediatric Endocrinologist, Department of Pediatrics, Alborz University of Medical Sciences, Karaj, Iran; 2 Alborz University of Medical Sciences, Karaj, Iran; 3 Medical Genetics, Tehran University of Medical Sciences, Tehran, Iran

**Keywords:** Niemann-Pick type A, Acid sphingomyelinase, Hypotonia, Neurodegenerative disorder, Case report

## Abstract

**Background:**

Niemann-Pick type A (NP-A) is a congenital, hereditary disease caused by a deficiency in acid sphingomyelinase, a lysosomal enzyme. This deficiency results in an accumulation of sphingomyelin in lysosomes, leading to cellular apoptosis and ultimately to hepatosplenomegaly, neurodegenerative disorder and failure to thrive. Cherry-red spots in the macula and foamy cells in the bone marrow are other manifestations of the disease that help with diagnosis. Type A is a rare, untreatable disease with early manifestations and a poor prognosis, with newborns rarely surviving for 2–3 years.

**Case presentation:**

A 1-year-old Persian boy was referred to our clinic due to abdominal distention and poor weight gain. He was the first male offspring of consanguineous parents. Other findings were neurodevelopmental delay, hepatosplenomegaly, severe hypotonia, difficulty in breathing, and a slightly coarse face with an open mouth and protruding tongue. The initial diagnosis was clinical mucopolysaccharidosis (MPS) based on the coarse facial features, but further workup ruled out this inherited disorder. Enzyme histochemistry revealed that the level of acid sphingomyelinase was lower than normal. In the genetic study, next-generation sequencing of all coding exons and flanking intronic regions of the patient’s DNA demonstrated a homozygous c.682T>G variant in the SMPD1 gene. This variant was classified as a variant of unknown significance. Further evaluation of DNA extract from his parents and examined using Sanger sequencing showed a heterozygous c.682T>G variant in the SMPD1 gene of both parents.

**Conclusions:**

We describe a 1-year-old boy with neurodevelopmental delay, hepatosplenomegaly, and severe hypotonia. Further investigation demonstrated a new mutation for Niemann-Pick disease.

## Introduction

The Niemann-Pick diseases are a group of autosomal recessive diseases that are categorized as lysosomal lipid storage disorders. These diseases are currently categorized into two groups: (1) acid sphingomyelinase-deficient Niemann-Pick disease (ASM-deficient NPD), either type A or type B (NP-A or NP-B), resulting from mutations in the SMPD1 gene; and (2) Niemann-Pick disease type C (NP-C), which also includes type D, resulting from mutations in either the NPC1 or the NPC2 gene [1].

NP-A is a congenital, inherited disease caused by a deficiency in acid sphingomyelinase, a lysosomal enzyme. Loss of acid sphingomyelinase causes sphingomyelin to accumulate in the central nervous system, liver, spleen, lungs, and bone marrow [2]. Acid sphingomyelinase deficiency (ASMD) results from mutations in the SMPD1 gene, and is categorized into an infantile neurovisceral form (historically referred to as type A), a chronic visceral form (historically referred to as type B), as well as an intermediate chronic neurovisceral form (or type A/B). Type A is a rare autosomal recessive disorder caused by mutations in the SMPD1 gene located on bands 11p15.1–p15.4. It is a neurodegenerative disease with neonatal manifestations that lead to death in early childhood [3]. The neonatal period is usually normal in these patients, with the most common symptoms in the first months of life being vomiting or diarrhea (or both). Neurological examination often reveals no abnormalities until the age of 5–10 months. Patients subsequently present with hypotonia, progressive loss of acquired motor skills, loss of interest in their surroundings, and reduced spontaneous movements. They also show slowed nerve conduction velocity. A cherry-red spot in the macula is a typical feature of patients with NP-A, but it is typically not present until an advanced stage when the initial axial hypotonia is then accompanied by bilateral pyramidal signs. Patients will show increasing spasticity and, consequently, abolished deep tendon reflexes. Seizures may occur but are not a major sign. The child regularly becomes cachectic. Recurrent respiratory infections are a common complication. Most patients die before 2 to 3 years of age, mostly from respiratory failure following pulmonary infection [1]. Patients with NP-A are diagnosed with hepatosplenomegaly, failure to thrive, hypotonia, and a cherry spot in the macula, and their life span is very short (< 3 years)[4].

NP-B is a relatively less severe disease than NP-A and is characterized by hepatosplenomegaly, thrombocytopenia, interstitial lung disease, and dyslipidemia. Most patients with NP-B have little or no neurologic involvement, so some patients might survive until adulthood [5].

## Case presentation

The Persian patient was born through cesarean section at 38 weeks with a birth weight of 3100 g. There were no complications with the delivery. He was the first child of Iranian consanguineous parents. At the age of 9 months, his pediatrician referred him to our outpatient clinic due to abdominal distention and developmental delay. Hypotonia and developmental delay of gross motor skills, expressive language, and social skills were the main concerns. At the time of referral, the child could not sit on his own, raise his head, crawl, or speak. However, he could smile. Other disturbing symptoms were frequent vomiting, failure to thrive, recurrent respiratory infections, irritability, and sleep disturbance.

### Clinical findings

Physical examination revealed global neurodevelopmental delay, hepatosplenomegaly, severe hypotonia, difficulty in breathing, and a slightly coarse facial appearance with an open mouth and protruding tongue.

### Timeline

At the age of 9 months, he suffered from hypotonia and delayed development of gross motor skills, expressive language, and social skills. He could not speak, sit, raise his head, or crawl. At the age of 1 year and 10 months, he was diagnosed based on the results of a genetic study. At the age of 2 years, the patient developed recurrent seizures.

### Diagnostic assessment

Based on the initial manifestation, especially the coarse facial features, we initially suspected mucopolysaccharidosis (MPS) or mucolipidosis; however, the results of an enzyme assay ruled MPS out. Ophthalmological examination revealed the presence of cherry-red spots in the macula. Subsequent enzyme histochemistry of a dried blood spot for evaluation of the acid sphingomyelinase level revealed a level of 11 nmol/L per 24 h (normal value: 200–2500 nmol/L per 24 h). In the genetic study, we performed next-generation sequencing of all coding exons and flanking intronic regions of the patients’s DNA. This study showed a novel mutation of the SMPD1 gene, namely, a homozygous variant, identified as c.682T>G, p. Cys228Gly, in exon 2 of the SMPD1 gene (NM_000543).

### Therapeutic intervention

Niemann-Pick type A is a disease with a poor prognosis for which there is no specific treatment. We treated out patient with omega 3, levetiracetam, and levothyroxine for symptomatic therapy.

### Follow-up and outcomes

At the age of 2 years, the patient developed recurrent seizures. At this stage of the disease, he could not smile any longer, and he could not eat or drink. The patient was also diagnosed as suffering from primary hypothyroidism.

## Discussion

Storage diseases are a group of rare inherited metabolic disorders that are defined by the accumulation of ungraded molecules due to a defect in a specific enzyme. They are classified based on the site of aggregation, the type of substrate, and their clinical manifestations [6]. MPS, GM1-gangliosidosis, mucolipidosis (ML), oligosaccharidosis, Pompe disease, Gaucher disease, Fabry disease, Niemann-Pick diseases, and neuronal ceroid lipofuscins are examples of lysosomal storage diseases [6]. In some of these disorders, a coarse facial feature is one of the main signs (such as in MPS and ML) [7, 8]; however, some other storage disorders are largely indistinguishable based on the patient’s appearance (e.g., Gaucher and Niemann-Pick diseases) [6, 9]. Our patient, who was ultimately diagnosed with NP-A, did have slightly coarse facial features in addition to the other clinical manifestations of this disease. Some facial dysmorphia is not entirely unusual in patients with NP-A. In the present case, hypothyroidism may also have played a role in his appearance.

Failure to thrive, which may lead to the discovery of usually prominent hepatosplenomegaly since early life in patients, is the first step towards a diagnosis of Niemann-Pick diseases. Delay in reaching developmental motor milestones from the age of 8–9 months onwards and central hypotonia constitute the first neurological symptoms, which become more evident between the ages of 1 and 2 years. The subsequent course of the disorder includes a loss of acquired motor skills, proportionally less marked mental regression, followed by spasticity with pyramidal tract involvement [1]. Our patient suffered from frequent vomiting, failure to thrive, hepatosplenomegaly, hypotonia, and delayed gross motor development in the first months of his life.

Cherry-red spots in the macula are a typical feature in NP-A, and these were also present in our patient. Seizures and recurrent respiratory infections are also common complications, which also occurred in our patients as well [10].

During pregnancy, fetal hydrops (rapidly fatal) or fetal ascites can occur. In about 40% of patients, a prolonged neonatal cholestatic icterus is present in association with progressive hepatosplenomegaly [1]. However, our patient had a normal fetal period and was a normal neonate.

Different mutations of the genes associated with the Niemann-Pick disorders have been identified. Recent investigations have also identified a number of new ones. However, but it is possible to determine an association between Niemann-Pick disorders and novel pathologic mutations that have been previously classified as variants of unknown significance [11]. This is the case in our patient. The genetic study of our patient’s DNA revealed a homozygous variant, identified as c.682T>G, p. Cys228Gly, in exon 2 of the SMPD1 gene (NM_000543). Sanger sequencing of both parent’s DNA showed a heterozygous variant of c.682T>G, p. Cys228Gly in exon 2 of the SMPD1 gene in both parents, confirming the homozygous state in the patient.

There is no specific treatment for NP-A and the prognosis is poor. However, Diaz *et al.* published an article that reported the promising results of clinical trials for specific treatment of NP-B by enzyme replacement therapy [12]. Wasserstein *et al.* observed five adult patients with chronic ASMD and found that treatment with olipudase alfa for 30 months is well tolerated and clinically effective [13]. However, acid sphingomyelinase cannot cross the blood-brain barrier, necessitating other strategies for patients with NP-A. To date, no proposed treatment has reached the stage of clinical trials, and thus the management of patients remains symptomatic.

The advances made in the treatment of inborn errors of metabolism in recent years, including enzyme replacement therapy, substrate deprivation, pharmacological chaperone therapy, the transplantation of stem cells, and gene therapy, have raised hopes for the development of future treatments [2, 5, 6, 8, 9].

In conclusion, the patient was the offspring of consanguineous parents and both parents were heterozygote carriers of the same mutation. This situation raises the risk for future pregnancies, and we recommend the parents receive genetic counseling.

## Conclusions

In this study we describe a 1-year-old boy presenting with neurodevelopmental delay, hepatosplenomegaly and severe hypotonia. Further investigation revealed a homozygous variant, identified as c.682T>G, p. Cys228Gly, in exon 2 of the SMPD1 gene (NM_000543) of our patient. This is a new mutation for NP-A.

## Data Availability

Not applicable.
